# 5-Iodo-7-methyl-3-methyl­sulfinyl-2-phenyl-1-benzofuran

**DOI:** 10.1107/S1600536808014694

**Published:** 2008-05-21

**Authors:** Hong Dae Choi, Pil Ja Seo, Byung Ki Kim, Byeng Wha Son, Uk Lee

**Affiliations:** a Department of Chemistry, Dongeui University, San 24 Kaya-dong Busanjin-gu, Busan 614-714, Republic of Korea; b Department of Molecular Biology, Dongeui University, San 24 Kaya-dong Busanjin-ku, Busan 614-714, Republic of Korea; c Department of Chemistry, Pukyong National University, 599-1 Daeyeon 3-dong Nam-gu, Busan 608-737, Republic of Korea

## Abstract

The title compound, C_16_H_13_IO_2_S, was prepared by the oxidation of 5-iodo-7-methyl-3-methyl­sulfanyl-2-phenyl-1-benzofuran with 3-chloro­peroxy­benzoic acid. The phenyl ring makes a dihedral angle of 27.17 (9)° with the plane of the benzofuran fragment, with the O atom and the methyl group of the methyl­sulfinyl substituent lying on opposite sides of this plane. The crystal structure exhibits inter­molecular C—H⋯I inter­actions, and an I⋯O halogen bond of 3.107 (2) Å with a nearly linear C—I⋯O angle of 173.73 (6)°.

## Related literature

For the crystal structures of similar 5-halo-3-methyl­sulfinyl-2-phenyl-1-benzofuran compounds, see: Choi *et al.* (2007*a* ▶,*b* ▶). For a review of halogen bonding, see: Politzer *et al.* (2007 ▶).
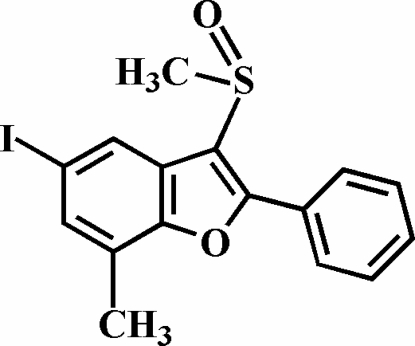


## Experimental

### 

#### Crystal data

C_16_H_13_IO_2_S
*M* *_r_* = 396.22
Monoclinic, 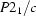

*a* = 10.385 (5) Å
*b* = 17.174 (8) Å
*c* = 8.943 (4) Å
β = 112.847 (7)°
*V* = 1469.9 (12) Å^3^
*Z* = 4
Mo *K*α radiation
μ = 2.32 mm^−1^
*T* = 173 (2) K
0.40 × 0.20 × 0.20 mm

#### Data collection

Bruker SMART CCD diffractometer
Absorption correction: multi-scan (*SADABS*; Sheldrick, 2000 ▶) *T*_min_ = 0.572, *T*_max_ = 0.623
11429 measured reflections
2877 independent reflections
2706 reflections with *I* > 2σ(*I*)
*R*_int_ = 0.032

#### Refinement

*R*[*F* ^2^ > 2σ(*F* ^2^)] = 0.020
*wR*(*F* ^2^) = 0.053
*S* = 1.06
2877 reflections
183 parameters
H-atom parameters constrained
Δρ_max_ = 0.39 e Å^−3^
Δρ_min_ = −0.66 e Å^−3^

### 

Data collection: *SMART* (Bruker, 2001 ▶); cell refinement: *SAINT* (Bruker, 2001 ▶); data reduction: *SAINT*; program(s) used to solve structure: *SHELXS97* (Sheldrick, 2008 ▶); program(s) used to refine structure: *SHELXL97* (Sheldrick, 2008 ▶); molecular graphics: *ORTEP-3* (Farrugia, 1997 ▶) and *DIAMOND* (Brandenburg, 1998 ▶); software used to prepare material for publication: *SHELXL97*.

## Supplementary Material

Crystal structure: contains datablocks global, I. DOI: 10.1107/S1600536808014694/zl2118sup1.cif

Structure factors: contains datablocks I. DOI: 10.1107/S1600536808014694/zl2118Isup2.hkl

Additional supplementary materials:  crystallographic information; 3D view; checkCIF report

## Figures and Tables

**Table 1:** Hydrogen-bond geometry (Å, °)

*D*—H⋯*A*	*D*—H	H⋯*A*	*D*⋯*A*	*D*—H⋯*A*
C3—H3⋯I^i^	0.95	3.06	3.954 (3)	157

Symmetry code: (i) 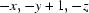.

**Fig. 1.**
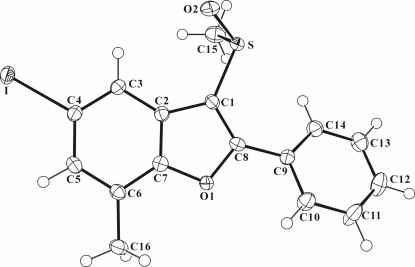
The molecular structure of the title compound, showing displacement ellipsoids drawn at the 50% probability level.

**Fig. 2.**
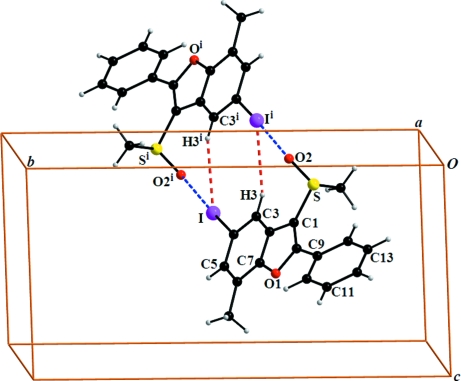
C—H···I interaction and I···O halogen bond (dotted lines) in the title compound. [Symmetry code: (i) -x, -y+1, -z.]

## supplementary crystallographic information

### Comment

This work is related to our previous communications on the synthesis and structure of 5-halo-3-methylsulfinyl-2-phenyl-1-benzofuran analogues, viz. 5-bromo-3-methylsulfinyl-2-phenyl-1-benzofuran (Choi *et al.*, 2007*a*) and 5-iodo-3-methylsulfinyl-2-phenyl-1-benzofuran (Choi *et al.*, 2007*b*). Here we report the crystal structure of the title compound, 5-iodo-7-methyl-3-methylsulfinyl-2-phenyl-1-benzofuran (Fig. 1).

The benzofuran unit is essentially planar, with a mean deviation of 0.013 Å from the least-squares plane defined by the nine constituent atoms. The phenyl ring (C9-C14) makes a dihedral angle of 27.17 (9)° with the plane of the benzofuran fragment. The molecular packing (Fig. 2) is stabilized by intermolecular C—H···I interactions (Table 1), and by an I···O halogen bond (Politzer *et al.*,  2007) between the iodine atom and the oxygen of a neighbouring S═O unit, with an I···O2^i^ distance of 3.107 (2) Å (symmetry code as in Fig. 2).

### Experimental

77% 3-chloroperoxybenzoic acid (247 mg, 1.1 mmol) was added in small portions to a stirred solution of 5-iodo-7-methyl-3-methylsulfanyl-2-phenyl-1-benzofuran (380 mg, 1.0 mmol) in dichloromethane (30 ml) at 273 K. After being stirred for 4 h at room temperature, the mixture was washed with saturated sodium bicarbonate solution and the organic layer was separated, dried over magnesium sulfate, filtered and concentrated in vacuum. The residue was purified by column chromatography (hexane-ethyl acetate, 1:1 v/v) to afford the title compound as a colorless solid [yield 79%, m.p. 433-434 K; R_f_ = 0.51 (hexane-ethyl acetate, 1:1 v/v)]. Single crystals suitable for X-ray diffraction were prepared by evaporation of a solution of the title compound in tetrahydrofuran at room temperature. Spectroscopic analysis: ^1^H NMR (CDCl_3_, 400 MHz) δ 2.53 (s, 3H), 3.11 (s, 3H), 7.49-7.58 (m, 4H), 7.83 (dd, J = 8.04 Hz and 1.84 Hz, 2H), 8.39 (s, 1H); EI-MS 396 [M^+^].

### Refinement

All H atoms were geometrically positioned and refined using a riding model, with C-H = 0.95 Å for aromatic H atoms, 0.98 Å for methyl H atoms, respectively, and with Uiso(H) = 1.2Ueq(C) for aromatic H atoms and 1.5Ueq(C) for methyl H atoms. The highest peak in the difference map is 0.98 Å from I and the largest hole is 0.92 Å from I.

### Crystal data

**Table:**

C_16_H_13_IO_2_S	*F*_000_ = 776
*M**_r_* = 396.22	*D*_x_ = 1.790 Mg m^−^^3^
Monoclinic, *P*2_1_/*c*	Mo *K*α radiation λ = 0.71073 Å
Hall symbol: -P 2ybc	Cell parameters from 9956 reflections
*a* = 10.385 (5) Å	θ = 2.1–28.4º
*b* = 17.174 (8) Å	µ = 2.32 mm^−^^1^
*c* = 8.943 (4) Å	*T* = 173 (2) K
β = 112.847 (7)º	Block, colorless
*V* = 1469.9 (12) Å^3^	0.40 × 0.20 × 0.20 mm
*Z* = 4	

### Data collection

**Table:**

Bruker SMART CCD diffractometer	2877 independent reflections
Radiation source: fine-focus sealed tube	2706 reflections with *I* > 2σ(*I*)
Monochromator: graphite	*R*_int_ = 0.032
Detector resolution: 10.0 pixels mm^-1^	θ_max_ = 26.0º
*T* = 173(2) K	θ_min_ = 2.4º
φ and ω scans	*h* = −12→12
Absorption correction: multi-scan(SADABS; Sheldrick, 2000)	*k* = −21→21
*T*_min_ = 0.572, *T*_max_ = 0.623	*l* = −11→11
11429 measured reflections	

### Refinement

**Table:**

Refinement on *F*^2^	Secondary atom site location: difference Fourier map
Least-squares matrix: full	Hydrogen site location: inferred from neighbouring sites
*R*[*F*^2^ > 2σ(*F*^2^)] = 0.020	H-atom parameters constrained
*wR*(*F*^2^) = 0.053	*w* = 1/[σ^2^(*F*_o_^2^) + (0.0278*P*)^2^ + 0.8832*P*] where *P* = (*F*_o_^2^ + 2*F*_c_^2^)/3
*S* = 1.07	(Δ/σ)_max_ = 0.001
2877 reflections	Δρ_max_ = 0.39 e Å^−^^3^
183 parameters	Δρ_min_ = −0.66 e Å^−^^3^
Primary atom site location: structure-invariant direct methods	Extinction correction: none

### Special details

**Table:**

Geometry. All esds (except the esd in the dihedral angle between two l.s. planes) are estimated using the full covariance matrix. The cell esds are taken into account individually in the estimation of esds in distances, angles and torsion angles; correlations between esds in cell parameters are only used when they are defined by crystal symmetry. An approximate (isotropic) treatment of cell esds is used for estimating esds involving l.s. planes.
Refinement. Refinement of F^2^ against ALL reflections. The weighted R-factor wR and goodness of fit S are based on F^2^, conventional R-factors R are based on F, with F set to zero for negative F^2^. The threshold expression of F^2^ > 2sigma(F^2^) is used only for calculating R-factors(gt) etc. and is not relevant to the choice of reflections for refinement. R-factors based on F^2^ are statistically about twice as large as those based on F, and R- factors based on ALL data will be even larger.

### Fractional atomic coordinates and isotropic or equivalent isotropic displacement parameters (Å^2^)

**Table:**

	*x*	*y*	*z*	*U*_iso_*/*U*_eq_	
I	−0.043288 (13)	0.558358 (8)	0.210981 (16)	0.02333 (7)	
S	0.36162 (5)	0.29269 (3)	0.14788 (6)	0.02051 (11)	
O1	0.50724 (14)	0.38231 (8)	0.59241 (16)	0.0184 (3)	
O2	0.30733 (17)	0.35243 (9)	0.01551 (18)	0.0280 (3)	
C1	0.3955 (2)	0.34115 (11)	0.3340 (2)	0.0186 (4)	
C2	0.3154 (2)	0.40433 (12)	0.3616 (2)	0.0185 (4)	
C3	0.1901 (2)	0.44237 (11)	0.2685 (3)	0.0199 (4)	
H3	0.1385	0.4291	0.1581	0.024*	
C4	0.1451 (2)	0.50027 (12)	0.3454 (2)	0.0203 (4)	
C5	0.2211 (2)	0.52118 (12)	0.5084 (2)	0.0211 (4)	
H5	0.1866	0.5614	0.5556	0.025*	
C6	0.3460 (2)	0.48423 (11)	0.6021 (2)	0.0195 (4)	
C7	0.3883 (2)	0.42659 (12)	0.5218 (2)	0.0182 (4)	
C8	0.5095 (2)	0.32999 (11)	0.4756 (2)	0.0180 (4)	
C9	0.6257 (2)	0.27448 (12)	0.5307 (2)	0.0184 (4)	
C10	0.7494 (2)	0.29442 (13)	0.6599 (3)	0.0267 (5)	
H10	0.7591	0.3445	0.7082	0.032*	
C11	0.8579 (2)	0.24128 (14)	0.7177 (3)	0.0357 (6)	
H11	0.9417	0.2550	0.8061	0.043*	
C12	0.8452 (2)	0.16800 (14)	0.6474 (3)	0.0333 (5)	
H12	0.9199	0.1317	0.6875	0.040*	
C13	0.7229 (2)	0.14820 (13)	0.5185 (3)	0.0284 (5)	
H13	0.7144	0.0984	0.4693	0.034*	
C14	0.6129 (2)	0.20047 (12)	0.4608 (3)	0.0242 (4)	
H14	0.5287	0.1861	0.3738	0.029*	
C15	0.4267 (2)	0.50275 (13)	0.7788 (3)	0.0265 (5)	
H15A	0.4092	0.4624	0.8460	0.040*	
H15B	0.3967	0.5534	0.8043	0.040*	
H15C	0.5268	0.5046	0.8010	0.040*	
C16	0.2127 (2)	0.23678 (14)	0.1402 (3)	0.0310 (5)	
H16A	0.1375	0.2721	0.1369	0.046*	
H16B	0.2391	0.2037	0.2369	0.046*	
H16C	0.1803	0.2041	0.0428	0.046*	

### Atomic displacement parameters (Å^2^)

**Table:**

	*U*^11^	*U*^22^	*U*^33^	*U*^12^	*U*^13^	*U*^23^
I	0.01881 (9)	0.02267 (10)	0.02713 (10)	0.00497 (5)	0.00739 (7)	0.00177 (5)
S	0.0221 (3)	0.0235 (3)	0.0161 (2)	0.0048 (2)	0.00748 (19)	−0.00083 (18)
O1	0.0190 (7)	0.0184 (7)	0.0170 (6)	0.0028 (5)	0.0060 (6)	−0.0001 (5)
O2	0.0330 (9)	0.0325 (8)	0.0175 (7)	0.0065 (7)	0.0088 (6)	0.0050 (6)
C1	0.0197 (9)	0.0193 (10)	0.0179 (9)	0.0009 (8)	0.0084 (8)	−0.0004 (7)
C2	0.0194 (9)	0.0184 (9)	0.0193 (9)	0.0006 (8)	0.0094 (8)	0.0015 (7)
C3	0.0199 (10)	0.0203 (10)	0.0187 (10)	0.0017 (8)	0.0067 (8)	0.0017 (7)
C4	0.0170 (9)	0.0227 (10)	0.0215 (10)	0.0023 (8)	0.0077 (8)	0.0038 (8)
C5	0.0248 (10)	0.0186 (10)	0.0226 (10)	0.0023 (8)	0.0123 (9)	−0.0007 (8)
C6	0.0233 (10)	0.0180 (9)	0.0193 (10)	−0.0003 (8)	0.0105 (8)	0.0006 (7)
C7	0.0185 (10)	0.0169 (9)	0.0194 (10)	−0.0004 (8)	0.0075 (8)	0.0025 (7)
C8	0.0205 (10)	0.0174 (9)	0.0181 (9)	−0.0016 (8)	0.0096 (8)	−0.0008 (7)
C9	0.0177 (9)	0.0206 (10)	0.0186 (9)	0.0003 (8)	0.0090 (8)	0.0040 (7)
C10	0.0219 (11)	0.0215 (10)	0.0324 (12)	−0.0011 (9)	0.0057 (9)	−0.0006 (9)
C11	0.0186 (11)	0.0308 (12)	0.0452 (14)	0.0026 (9)	−0.0013 (10)	0.0021 (10)
C12	0.0233 (11)	0.0274 (12)	0.0478 (14)	0.0080 (9)	0.0121 (11)	0.0091 (10)
C13	0.0324 (12)	0.0209 (10)	0.0338 (12)	0.0036 (9)	0.0150 (10)	0.0013 (9)
C14	0.0251 (11)	0.0232 (10)	0.0234 (10)	0.0010 (9)	0.0086 (9)	−0.0003 (8)
C15	0.0320 (12)	0.0269 (11)	0.0190 (10)	0.0020 (9)	0.0080 (9)	−0.0031 (8)
C16	0.0315 (12)	0.0311 (12)	0.0278 (11)	−0.0070 (10)	0.0087 (10)	−0.0055 (9)

### Geometric parameters (Å, °)

**Table:**

I—C4	2.107 (2)	C8—C9	1.465 (3)
I—O2^i^	3.107 (2)	C9—C10	1.396 (3)
S—O2	1.501 (2)	C9—C14	1.400 (3)
S—C1	1.770 (2)	C10—C11	1.385 (3)
S—C16	1.799 (2)	C10—H10	0.9500
O1—C7	1.377 (2)	C11—C12	1.390 (4)
O1—C8	1.385 (2)	C11—H11	0.9500
C1—C8	1.370 (3)	C12—C13	1.386 (3)
C1—C2	1.446 (3)	C12—H12	0.9500
C2—C7	1.389 (3)	C13—C14	1.385 (3)
C2—C3	1.404 (3)	C13—H13	0.9500
C3—C4	1.389 (3)	C14—H14	0.9500
C3—H3	0.9500	C15—H15A	0.9800
C4—C5	1.409 (3)	C15—H15B	0.9800
C5—C6	1.394 (3)	C15—H15C	0.9800
C5—H5	0.9500	C16—H16A	0.9800
C6—C7	1.391 (3)	C16—H16B	0.9800
C6—C15	1.507 (3)	C16—H16C	0.9800
			
C4—I—O2^i^	173.73 (6)	C10—C9—C8	119.45 (19)
O2—S—C1	107.27 (10)	C14—C9—C8	121.16 (18)
O2—S—C16	106.30 (11)	C11—C10—C9	120.1 (2)
C1—S—C16	98.10 (10)	C11—C10—H10	120.0
C7—O1—C8	106.69 (15)	C9—C10—H10	120.0
C8—C1—C2	107.15 (17)	C10—C11—C12	120.5 (2)
C8—C1—S	126.05 (16)	C10—C11—H11	119.8
C2—C1—S	126.59 (15)	C12—C11—H11	119.8
C7—C2—C3	119.48 (19)	C13—C12—C11	119.6 (2)
C7—C2—C1	105.27 (17)	C13—C12—H12	120.2
C3—C2—C1	135.23 (19)	C11—C12—H12	120.2
C4—C3—C2	116.85 (19)	C14—C13—C12	120.5 (2)
C4—C3—H3	121.6	C14—C13—H13	119.7
C2—C3—H3	121.6	C12—C13—H13	119.7
C3—C4—C5	122.24 (19)	C13—C14—C9	120.0 (2)
C3—C4—I	118.20 (15)	C13—C14—H14	120.0
C5—C4—I	119.56 (15)	C9—C14—H14	120.0
C6—C5—C4	121.56 (19)	C6—C15—H15A	109.5
C6—C5—H5	119.2	C6—C15—H15B	109.5
C4—C5—H5	119.2	H15A—C15—H15B	109.5
C5—C6—C7	114.84 (18)	C6—C15—H15C	109.5
C5—C6—C15	122.91 (19)	H15A—C15—H15C	109.5
C7—C6—C15	122.21 (19)	H15B—C15—H15C	109.5
O1—C7—C2	110.71 (17)	S—C16—H16A	109.5
O1—C7—C6	124.27 (18)	S—C16—H16B	109.5
C2—C7—C6	125.00 (19)	H16A—C16—H16B	109.5
C1—C8—O1	110.17 (17)	S—C16—H16C	109.5
C1—C8—C9	134.83 (18)	H16A—C16—H16C	109.5
O1—C8—C9	114.93 (16)	H16B—C16—H16C	109.5
C10—C9—C14	119.33 (19)		
			
O2—S—C1—C8	−138.12 (18)	C5—C6—C7—O1	−179.08 (18)
C16—S—C1—C8	111.9 (2)	C15—C6—C7—O1	−1.3 (3)
O2—S—C1—C2	36.1 (2)	C5—C6—C7—C2	−0.8 (3)
C16—S—C1—C2	−73.9 (2)	C15—C6—C7—C2	176.9 (2)
C8—C1—C2—C7	−0.4 (2)	C2—C1—C8—O1	0.0 (2)
S—C1—C2—C7	−175.49 (15)	S—C1—C8—O1	175.08 (14)
C8—C1—C2—C3	−179.1 (2)	C2—C1—C8—C9	176.7 (2)
S—C1—C2—C3	5.9 (3)	S—C1—C8—C9	−8.2 (3)
C7—C2—C3—C4	−1.1 (3)	C7—O1—C8—C1	0.5 (2)
C1—C2—C3—C4	177.4 (2)	C7—O1—C8—C9	−176.98 (16)
C2—C3—C4—C5	0.8 (3)	C1—C8—C9—C10	157.6 (2)
C2—C3—C4—I	−179.53 (14)	O1—C8—C9—C10	−25.8 (3)
C3—C4—C5—C6	−0.5 (3)	C1—C8—C9—C14	−25.2 (3)
I—C4—C5—C6	179.82 (15)	O1—C8—C9—C14	151.38 (18)
C4—C5—C6—C7	0.5 (3)	C14—C9—C10—C11	0.0 (3)
C4—C5—C6—C15	−177.3 (2)	C8—C9—C10—C11	177.3 (2)
C8—O1—C7—C2	−0.7 (2)	C9—C10—C11—C12	0.4 (4)
C8—O1—C7—C6	177.71 (19)	C10—C11—C12—C13	0.0 (4)
C3—C2—C7—O1	179.63 (17)	C11—C12—C13—C14	−0.8 (4)
C1—C2—C7—O1	0.7 (2)	C12—C13—C14—C9	1.2 (3)
C3—C2—C7—C6	1.2 (3)	C10—C9—C14—C13	−0.8 (3)
C1—C2—C7—C6	−177.73 (19)	C8—C9—C14—C13	−178.01 (19)

Symmetry codes: (i) −*x*, −*y*+1, −*z*.

### Hydrogen-bond geometry (Å, °)

**Table:**

*D*—H···*A*	*D*—H	H···*A*	*D*···*A*	*D*—H···*A*
C3—H3···I^i^	0.95	3.06	3.954 (3)	157

Symmetry codes: (i) −*x*, −*y*+1, −*z*.
